# A public policy analysis with key stakeholders’ insights to understand India’s compliance with the WHO framework convention on tobacco control

**DOI:** 10.3332/ecancer.2022.1402

**Published:** 2022-05-26

**Authors:** Soumita Ghose, Aseem Mahajan, Soumitra Shankar Datta

**Affiliations:** 1 Department of Medical Administration and Policy, Tata Medical Center, New Town, Kolkata 700160, India; 2 Department of Palliative Care and Psycho-oncology, Tata Medical Center, Kolkata 700160, India; 3 MRC Clinical Trials Unit, Institute of Clinical Trials and Methodology, University College London, 90 High Holborn, London, UK; a https://orcid.org/0000-0003-0084-1283; b https://orcid.org/0000-0001-9060-7823; c https://orcid.org/0000-0003-1674-5093

**Keywords:** tobacco, policy analysis, cancer, oncology, LMIC, India, health policy, tobacco policy, FCTC, COTPA

## Abstract

**Background:**

Tobacco-related morbidity and mortality is a global public health challenge. India is the second largest consumer of tobacco in the world. The present paper synthesises the data from qualitative interviews of experts working in the field of tobacco control alongside a critical analysis of the national tobacco control policy of India.

**Methods:**

The research methods adopted for the present work included the following: 1) qualitative in-depth interview of experts and analysis of the qualitative data using thematic analysis; 2) searching existing literature and secondary data on the national tobacco policy and analysing the same using the methodological orientation of qualitative content analysis; and 3) health policy analysis of the national tobacco policy. Themes and sub-themes obtained from the two approaches were compared to generate meaning.

**Results:**

Nine experts (three women and six men) participated in the in-depth qualitative interviews from a variety of professional backgrounds (preventive oncology researcher, tobacco cessation specialist, public health expert, clinicians and human rights activists). The systematic and comprehensive literature search resulted in finding 14 research papers, reviews, policy documents and commentaries on the tobacco epidemic in India. The various themes that emerged from the qualitative interviews that found support from secondary data as well were: ‘*Conflicting policies of Government: Promoting tobacco production and at the same time restricting its use’, ‘Failure to shield from influence of tobacco industry’, ‘Demand reduction proposals through taxation and pricing’, ‘Legislation to protect from and reduce the harm of second-hand smoking’, ‘Health warning on packaging and labelling of tobacco products’, ‘Promotion and advertisement of tobacco products’, ‘Prohibiting production by and sale to minors’.*

**Discussion:**

The control of a tobacco epidemic has multiple structural and functional challenges embedded in the complexity of the public–private interfaces, socio-economic forces, conflicting interests of the stakeholders and diverse nature of the problem. Any intervention planned to reduce the tobacco usage at a population level needs to take these factors into consideration.

## Background

The widespread use of tobacco is one of the biggest global health challenges [1]. It has been recognised as a deterrent to the success of the United Nations Millennium Development Goals [2]. Tobacco is also the single largest cause of preventable death [1]. The most commonly used tobacco products are cigarettes that rapidly deliver nicotine to the brain after inhalation and also contain many other chemicals that are proven carcinogens [3]. Scientific studies show that all forms of tobacco can be lethal [1]. Although the overall use of smokeless tobacco has reduced in the past 10 years, it remains popular amongst women and all users who use more than one form of tobacco in India [4, 5]. A recent study estimated that the economic costs of tobacco use for the population aged 35 years and older was 27.7 billion dollars in 2017–18 [6]. More than half of the tobacco users are killed from tobacco-related illnesses [1]. A significant number of second-hand smokers suffer from fatal consequences, prenatal anomalies and chronic illnesses [1] killing an estimated 1.2 million of them each year globally [7].

### India on the map of the global tobacco epidemic

Low- and middle-income countries (LMICs) are home to more than 80% of the tobacco users of the world, and thus contribute significantly to tobacco-related mortality and morbidity [8]. Tobacco use is projected to kill one billion in the 21st century and 80% of these deaths will be from LMICs [9]. The widespread use of tobacco in the developing world is attributed to its low price, variations in form, lack of awareness on harmful effects, unregulated aggressive marketing by the tobacco industry and lack of public health policies advocating for its complete ban. India is home to approximately 266.8 million tobacco users and is the second largest consumer of tobacco in the world [8, 10]. India’s tobacco landscape is more complex due to the extensive production facilities and use of many smokeless forms of tobacco. Currently, it is estimated that 42.4% men, 14.2% women and 28.6% of all adults use tobacco in India. A vast majority of the users (87%) debut tobacco use before the age of 18 years [8]. Furthermore, according to the WHO Tobacco Atlas, 625,000 children between the age of 10 and 14 years use tobacco in some form in India [10]. Due to the massive use of smokeless tobacco which is often homemade, India has one of the highest rates of oral cancer in the world with an annual incidence as high as 14.8 per 100,000 among males [11]. Workers engaged in tobacco cultivation in India also suffer from significant disease burdens such as green tobacco sickness which is an acute form of nicotine toxicity [12]. The use of tobacco also significantly impacts the economic livelihood of low-income groups where 3% of the household budget may be spent on tobacco impacting on the poverty levels further [13].

The World Health Organization Framework Convention on Tobacco Control (WHO-FCTC) is known to be the world’s first public health treaty [14]. Enacted under the WHO, the treaty was developed in response to the globalisation of the tobacco epidemic acknowledging the pivotal role of international law in preventing disease and promoting global health. The primary objective of the treaty is to ‘protect present and future generations from the devastating health, social, environmental and economic consequences of tobacco consumption and exposure to tobacco smoke’. The treaty provides an actionable blueprint to the signatories mainly targeting to reduce the demand and supply of tobacco. With more than 180 signatories representing almost 90% of the world’s population [9], WHO-FCTC is the biggest global initiative in the history of tobacco control. After almost two decades of signing the treaty, India still holds a central place on the global tobacco problem. The long-term success of the treaty will rely on countries like India which is a major driver not only in terms of consumption of tobacco but also being the second leading world producer of leaf tobacco [15].

Post ratification of the WHO-FCTC treaty, the Indian government instituted the Cigarettes and Other Tobacco Products Act (COTPA) in the year 2003 [16]. Being the single largest legislation on tobacco control in the country, COTPA prohibits smoking in public places, controls advertisement of tobacco products, restricts sale of tobacco to minors and within 100 yards of educational institutions [16].

The number of published policy analysis addressing tobacco control in India is scarce even though there are a significant amount of analyses done in other LMICs such as China [17] and Brazil [18], making the need for a policy analysis stronger. We have published previously on the strengths and gaps in the national tobacco policy and contrasted this with the qualitative data obtained from young adult tobacco users [19]. The goal of the present paper is twofold: a) analyse India’s tobacco control policies considering the WHO-FCTC, identify barriers and gaps, b) reflect on key stakeholders’ perspectives and recommend opportunities for policy reforms.

## Methods

The methodology adapted for this paper will be described in five sections as follows: 1) methodology of interviewing experts and analysing qualitative data of expert opinions; 2) methodology for searching the secondary data and existing literature commenting on the tobacco policy; 3) qualitative methodology adapted for analysing the secondary data and existing literature commenting on the tobacco policy; 4) methodology adapted for the policy analysis of COTPA; and 5) juxtaposing the policy statements of COTPA and corresponding WHO-FCTC sections alongside qualitative data obtained from Sections 1 and 3. The study was approved by the institutional ethics committee of the Tata Medical Center, Kolkata (EC/TMC/66/16).

*The research questions examined in the paper are:*

1. What is the current understanding of the national law and policies implemented for tobacco control in India?
2. To what extent are WHO-FCTC measures incorporated in the national policies, legislations and the National Tobacco Control Program (NTCP) as reported by experts and stakeholders?
3. What are the gaps in the National Tobacco Control Program and tobacco control legislation in India as reported by experts and stakeholders?

**Figure 1.**
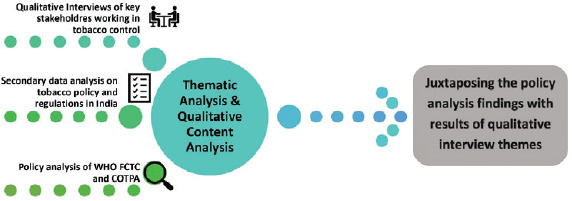
Summary diagram of the methodology.

**1) Methodology of interviewing experts and analysing qualitative data of expert opinions (** Figure 1 **)**

### Study setting

The study was conducted in the Tata Medical Center, Kolkata which is a not for profit cancer hospital in eastern India. The hospital treats patients from a wide catchment area in eastern India and the neighbouring countries of Bangladesh, Bhutan and Nepal. Tobacco-related cancers are common in India where this study was conducted.

### Researchers

The research team consisted of three experts, two of whom (SSD and SG) are trained in conducting qualitative research and have published qualitative research papers in peer reviewed international journals. SSD is a consultant psychiatrist working in the field of psycho-oncology for more than a decade. SG is a medical administrator with a public health background. The third member of the research team is an expert on medical policies and health policy implementation in India and has a formal qualification in medical management.

### Selection of participants

Participants were selected, using purposive sampling, from a wide geographical region of India covering north, south, east and western India.

### Inclusion criteria

Inclusion criteria for experts was based on their professional background of working on the tobacco epidemic as policy experts, clinicians, researchers and service providers helping rehabilitating children and adults affected by tobacco production and use.

### Exclusion criteria

Participants with known funding obtained from the tobacco industry were excluded and not approached for participation.

### Qualitative interviews

In-depth qualitative interviews were conducted by SG. Written informed consent was obtained prior to interviewing of participants. Due to the political nature of the project, all stakeholders were kept anonymous in the final results.

### Sample size

Sample size was determined by the principles of data saturation. The interviews were stopped when new themes stopped emerging from the interviews.

### Data analysis

The interview data were analysed using the framework of thematic analysis as suggested by Braun and Clarke [20]. The interviews were coded by two researchers (SG and SSD). A third researcher (AM) reviewed all the codes and helped to sort out any differences in coding between two coders. The steps of qualitative data analysis for the interviews were: (a) generating codes, (b) charting the data, (c) data synthesis, (d) generating basic themes and (e) generating global themes.

**2) Methodology for searching the secondary data and existing literature commenting on the tobacco policy**

Publicly available secondary data covering the timeframe of 2004 to present were accessed. Apart from the main WHO-FCTC treaty and India’s tobacco control law, COTPA, a database search was conducted for published academic sources. The search platform used for the research permitted cross-searching of major databases licensed by the University of Alberta including MEDLINE, CINAHL, Health Policy Reference Centre, Science Direct and Social Science Citation Index as well as other health, social science and interdisciplinary databases. Relevant key words for the search were ‘WHO-FCTC’ OR ‘Framework convention on tobacco control’ AND WHO AND ‘National tobacco control program’ OR ‘tobacco control’ AND India. Evidence to support analytic arguments are also drawn from grey literature and through interaction with key informants, actors or stakeholders engaged in tobacco control in India.

**3) Qualitative methodology adapted for analysing the secondary data and existing literature commenting on the tobacco policy**

The documents retrieved from the public domain using the above search strategy were analysed using the methodological orientation of qualitative content analysis (QCA) [21]. QCA is particularly suited for exploratory work and in generating meaning of data. Data analysis was done manually. The two researchers (SG and SSD) coded the data. As recommended in QCA, an initial coding frame was constructed based on the themes generated by the qualitative interviews and also content of the documents. The following steps were followed to build a coding frame as suggested by Schreier [21]: a) generating categories; b) defining categories; and c) revising the coding frame. Following this methodology, a coding frame was built by SG and SSD. Subsequently, the two coders (SG and SSD) independently divided the content of the documents, that were analysed, into the coding frame that went through a few iterations of modifications.

**4) Methodology of public policy analysis**

According to modern health policy analysis, policy reform is a political process that needs to take into consideration the reasons why policy outcomes failed to emerge. This portion of the analysis is based on the paradigm of policy thinking as discussed in the works of Walt and Gilson who analysed the policy world in its entirety in the form of a ‘thick description’ [22] and not based only on its content matter. This methodology considers the complexity of the milieu in which public health policies are set in today’s world. Public health policies often emerge amidst multiple conflicts and resistance during development. Public health policies rarely reach universal consensus of all the stakeholders. We have also followed a policy triangle as suggested by Walt and Gilson [22] in the present paper. This framework developed for the health sector had found its use in a wide array of health issues in different countries, especially LMICs. Grounded in political economic perspective, this framework critically reviews a policy from three major heads forming a triangle, namely, the i) context or the historical set up, ii) the content and iii) the process of formulation. In this framework, stakeholders or actors are also identified and their roles in the policy are critically analysed. The framework is fit for use retrospectively, which suits current work. Going by Gill Walt’s philosophy, this paper attempted to look at ‘what explains what happened’ rather than only looking at ‘what happened’ [23].

**5) Juxtaposing the policy statements of COTPA and corresponding WHO-FCTC sections alongside qualitative data obtained from 2 and 3**

The health policy analysis section primarily bases itself on publicly available secondary data on the country’s compliance to tobacco control measures through the implementation of COTPA, looking at the time frame from the year India signed the treaty to the present time. In addition, this study has a qualitative component where key informants and tobacco control advocates from the public health industry of India had been interviewed and their views on India’s legal framework versus the WHO-FCTC have been analysed and compared with the results of policy analysis. An interview guide was used which included both targeted questions related to WHO-FCTC and The Cigarettes and Other Tobacco Products (prohibition of advertisement and regulation of trade and commerce, production, supply and distribution) Act, 2003 (COTPA) and how the two compare and create India’s tobacco control landscape.

To summarise, this policy analysis is conducted with two very careful considerations. First, India’s tobacco use behaviour is unique in the world. One of the primary focuses of this report is analysing this uniqueness and understanding how it acts as a barrier instead of just comparing it with global practices. Second, a subject such as tobacco control gives rise to a wide array of political, economic and socio-cultural issues which can derive different analytical results depending upon the trajectory of analysis chosen. This report is formulated with a public health lens but it recognises the complex interplay that health has with its social determinants, human rights, cultural and economic environment and political will of a country. The study adhered to the COREC guidelines for qualitative research [24].

## Results

The results of this study stemmed from two sources; first, the qualitative interviews with experts leading to seven major themes, and second, the qualitative content analysis of the secondary data available on the topic of the tobacco epidemic in India.

**Figure 2.**
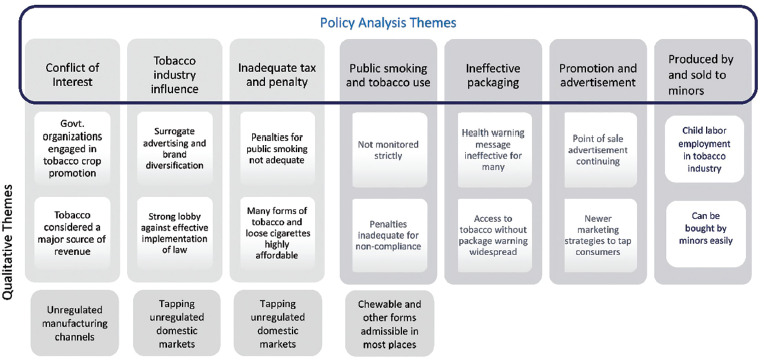
Coding diagram of thematic analysis and qualitative content analysis.

Nine experts participated in the in-depth qualitative interviews as part of the study (Table 1). The participants had a mix of gender (three women and six men) from a variety of professional backgrounds (preventive oncology researcher, tobacco cessation specialist, public health expert, clinicians and human rights activists) representing a wide geographical region of the country including Western India (Maharashtra), Eastern India (Kolkata), Southern India (Kerala) and Northern India (Delhi). The secondary data from the published literature from a selection of high-quality relevant papers commenting on the issue, from around the country, was used for the policy analysis. The systematic and comprehensive literature search conducted according to the methodology described earlier resulted in finding 16 sources of documents that included research papers, reviews, policy documents and commentaries that were relevant to our paper (Table 2). Eleven of these were specifically on India and the three others commented on India and the global tobacco epidemic. Qualitative content analysis was conducted on these documents and the themes generated are shown in Figure 2.

The seven main themes generated from the public policy analysis have been interwoven with the associated qualitative piece and written as one comprehensive theme (Figure 2).

The policy analysis piece compares the guidelines from WHO-FCTC and the legislations laid down in COTPA and serves as a gap analysis between the two; findings from this gap analysis are correlated with the themes generated from the interviews. The themes are rendered in this paper in association to the relevant sections of the WHO-FCTC and COTPA to demonstrate how the gaps in the policy compare to the themes generated from interviewing key stakeholders. The results section also includes the contextual placement of India in the global tobacco trade landscape to understand some of its policy decisions from a clearer perspective. Relevant excerpts from the qualitative interviews of experts have been provided against each main theme.

### Analysis of the content of the COTPA in light of WHO WHO-FCTC

This section compares India’s tobacco control policies with contextually relevant articles of the Framework Convention treaty by comparing both the documents in their content as well as through compliance and gaps reported in the published literature. It further analyses, the policy gaps through themes generated from the interviews.

**Theme 1.** Conflicting policies of government: promoting tobacco production and at the same time restricting its use.

Framework convention treaty	Policy gap with COTPA	Qualitative interview
**Article 3. Objective of the treaty** WHO-FCTC Article 3 summarises the primary objective of the treaty being ‘to protect present and future generations from the devastating health, social, environmental and economic consequences of tobacco consumption and exposure to tobacco smoke’. It urges governments to implement the framework at the national, regional and international level to reduce use and exposure of tobacco in the population.	The act intends to prohibit advertisement, regulation of trade and commerce and production supply and distribution of cigarettes and other tobacco products but Conflicting policies of the Government enable promoting enables Promoting tobacco production and at the same time restricting its use	*‘….there is no interdepartmental coordination, like tobacco control is managed by different departments like revenue department collects the money and trade and farmers ministry is promoting the tobacco board and supporting the tobacco industry and their mission is augmenting the services and supporting helping the development of the tobacco industry and the agricultural department is promoting tobacco cultivation and when different departments are working with different missions in terms of tobacco control and there is no coordinated policies adapted and they don’t work together……one department is suffering here to control tobacco where as another department is striving hard to promote tobacco…’ (In charge of a Tobacco cessation clinic in an Oncology Center, Kerala, India)*

Article 47 of the Indian constitution published in 1948 explicitly mentioned that the State will endeavour to prohibit intoxicating drinks and drugs unless the use was medicinal [35]. Tobacco was, however, treated as a major source of public revenue along with applying legal control on its use. The Indian parliament and lawmakers had been big advocates for tobacco control, but the tobacco industry took a firm stand indicating huge financial losses. This was the first evidence among many instances of exercising contradictory public policies. The Tobacco Act was enacted by the parliament in 1975 and the Indian Tobacco Board was also constituted under the Ministry of Commerce to develop the tobacco industry [37]. These conflicting forces historically delayed legislative changes towards tobacco control. After half a century of independence, the Cigarettes Act was passed in 2003 [25].

There are a few noted entities aiding and facilitating the production of tobacco in India. The Indian Tobacco Board and agricultural research institutes in different states in India are facilitating its sustained growth alongside several large business enterprises [35]. The Indian Tobacco Board also acted as a seed bank, a seed research body and gives loans to farmers [25]. Constituted in 1966, the Indian Tobacco Development Council aided in planning and execution of tobacco production and marketing. The bidi manufacturing industry is another big actor that is rapidly becoming an organised corporate sector. The thriving tobacco industry thus remains a major barrier to tobacco control. With a constantly growing sales turnover over the last decade, this industry is also a major employer. The industry spends millions of dollars in researching consumer behaviour, advertising and tapping new segments of the market [38]. While the Ministry of Health and Family Welfare focuses on tobacco control measures, the Ministry of Commerce and Industry promotes tobacco as a crop at the same time [25]. The tobacco industry also gets represented in government meetings as representatives of the food industry through brand diversification aiming to influence public health decisions taken in favour of tobacco control [25].

**Theme 2.** Failure to shield from influence of tobacco industry.

Framework convention treaty	Policy gap with COTPA	Qualitative interview
**Article 5.3** Protect tobacco policies from commercial and other vested interests of the tobacco industry in accordance with national law	Enables punishment for advertisement but fails to protect from industry influence	*‘What is very disturbing is the place of work the cancer centre that I am attached to, in the periphery there are half a dozen tobacco shops so it’s like you know we are not taking care of the prevention aspects...’ (Clinical Psychologist working in an NGO for Cancer Prevention, Mumbai, India)*

Article 5.3 of the WHO-FCTC treaty requires ‘the signatories to protect public health tobacco control policies from commercial and vested interests of the tobacco industry’ [14]. Article 5.3 is known to be the most widely debated and noncompliant section under the WHO-FCTC. Published and anecdotal evidence and the history of tobacco trade reaffirm the government’s conflicting roles in tobacco control in India with no national policy to protect public health interests from tobacco industry and to limit the interaction of government agencies with the tobacco industry therefore making implementation of article 5.3 ineffective [25, 36]. There are also reports of conflict of interest existing within administrative positions where individuals hold positions or stakes both in tobacco companies and within bodies responsible for controlling of the tobacco epidemic [25, 36]. Tobacco continues to be a prime contributor to the Indian economy. Its potential for generating consistent revenue, employment for millions and the capacity of export to the whole world had made the tobacco industry a highly sought-after trade in India [35]. India currently produces approximately 730 million kg of dry tobacco annually [39]. This makes India the second largest tobacco producer and exporter (260 million kg) globally [39]. Over the years, the tobacco industry has grown from being only a cash crop to a manufacturing industry with huge commercial interest due to the combination of strong prices, high domestic consumption and demand for export. Indian tobacco is exported to more than 100 countries currently [35, 39]. Dominated by multinational corporations, the organised sections of this industry play a huge role in supporting its growth. This poses a great challenge for public health policymakers. There is also an unrecognised domestic market other than cigarettes and bulk of production is done in this category, while cigarettes account for only 30% of production [35]. However, domestic production data for these forms are not easily available indicating inability of the system to monitor this unregulated domestic market [40]. Despite growing public health concerns, tobacco industry in India is thriving due to this employment and revenue generating capabilities. It was able to lobby against implementation of 85% package warning on cigarette packets and, therefore, leading to delay in implementation of the same [25]. The production of bidi is a highly labour-intensive activity [35]. The leaf growing industry employs people in the magnitude of many millions and mostly from regional and rural communities with little or no education, the unorganised sector employs millions of tribal indigenous people [40]. Tobacco is used in a wide variety of ways in India such as smoking, chewing, applying, sucking and gargling; for each of these types, a wide range of tobacco products are available [26]. Often, they are locally manufactured or prepared by the seller or user. Many of these do not pass through any formal production, supply and distribution chain, thus remaining largely unrecognised and unregulated. These alternative forms are also cheaper leading to high rates as well as early debut of use in lower socio-economic classes [26, 38]. While the use of smokeless tobacco (SLTs) is the second most common form in the country, legislative measures historically have focused on smoked tobacco only, therefore, allowing the tobacco industry to continue expanding its market [26]. In addition, tobacco companies have historically attempted and continued to invest in research on nicotine replacement therapy (NRT) products and their variations in form; therefore, tapping the consumer markets who are trying to quit smoking, often this has led to users opting for both tobacco products as well as NRTs [41].

**Theme 3.** Demand reduction proposals through taxation and pricing.

Framework convention treaty	Policy gap with COTPA	Qualitative interview
**Article 6.** ‘implementing tax policies and, where appropriate, price policies, on tobacco products so as to contribute to the health objectives aimed at reducing tobacco consumption’	Inadequate taxation to act as a deterrent to smoking	*‘…they (students) can buy it anywhere with their pocket money …’ (Clinical Psychologist working in an NGO for Cancer Prevention, Mumbai, India)*

Article 6 of the WHO-FCTC treaty recommends implementation of taxation and pricing policies for demand reduction [14]. Currently, the Indian law does not specify taxation and pricing rules for any forms of tobacco specifically and taxation is focused on cigarettes allowing SLT manufacturers to use legal loopholes to continue production and distribution through unregulated channels [16, 28]. Studies report a decline in India’s tobacco taxation compliance [42]. Tobacco products are taxed in the country at two levels, the centre (excise duty) and the state. Current prices of these products are highly affordable for the general public. The cheapest retail sale price (including tax) for a pack of 20 cigarettes is INR. 38 (US$ 0.57). The most popular brand cost INR 106 (US$ 1.59) for a pack of 20 cigarettes [43, 44]. The smokeless forms, which are consumed more than cigarettes, are priced much lower [45]. A packet of 20 bidis would cost only INR 10 (US$ 0.15) and a pack of chewing tobacco would be INR 7 (US$ 0.1) [43, 44]. Furthermore, cigarettes are taxed at 60.39% of total retail price, but bidi is taxed at 19.74% only and chewable tobacco is taxed at 50.98% [43, 44]. This does not reflect a good policy choice as the current estimated users of bidis are much higher than cigarette smokers in the country [13]. There are other legal gaps such as manufacturers producing less than 2 million pieces annually do not have to pay any excise duty. Due to this, reportedly 90% of the chewing tobacco manufacturers escape excise duties by reporting only 10% of their production [46]. India is the only country which has length wise and filter-based excise duty put on cigarettes. The tobacco companies have invented ‘mini’ and ‘non-filter’ cigarettes to pay less tax due to the smaller size and low filter [47]. Overall, cigarettes in India are not less affordable than before [43, 44], and the tax and prices imposed did not attain the goal of demand reduction significantly.

**Theme 4.** Legislation to protect from and reduce the harm of second-hand smoking.

Framework convention treaty	Policy gap with COTPA	Policy gap and qualitative theme
**Article 4.** ‘effective legislative, executive, administrative or other measures should be contemplated at the appropriate governmental level to protect all persons from exposure to tobacco smoke’. **Article 8.** ‘…implementation of effective legislative, executive, administrative and/or other measures, providing for protection from exposure to tobacco smoke in indoor workplaces, public transport, indoor public places and, as appropriate, other public places’.	Focus on banning public smoking as opposed to public use of any form of tobacco. Definition of ‘public smoking’ renders legal loopholes. Responsibility is on general public to report violations.	*‘….they continue to say that we have a designated smoking area, only in that area they can smoke but 100 people if they stand there and smoke and 200 people just walking by the side of that, there is no logic of having a non-smoking area and smoking area,…….and that has to be completely removed there is no concept of designated smoking area when we talk about a ban on smoking in public places,….. and they also say that if it is an open space smoking is allowed so there is a bus stand and it is an open space and majority of the cases there are 5 -6 people and children and they are standing there…’ (In charge of tobacco cessation clinic in an Oncology Center, Kerala, India)*

Framework Convention Article 8 recommends providing protection against exposure to second hand smoke in public places [14]. The COTPA bans smoking in most public places such as workplace, hospitals, educational institutions, etc. [16]. But 3 out of 10 adults working indoors are exposed to second hand smoke [27]. As per the law, smoking rooms are to be provided in airports, hotels and restaurants and other public places housing more than 30 people [16]. However, penalty for smoking in a public place is only INR 200 (US$3) which is affordable by most and is hardly of any deterrent value. It is also the responsibility of general population to report defaulters; therefore, making the surveillance of smoking in public places less stringent. However, definition and interpretation of a ‘public’ space in a populous country like India can be confusing leading to noncompliance and mismatched expectations between the treaty and the law [48].

Majority of the population uses chewable forms of tobacco often in public places and indoors, without having to comply with any stringent norms [49]. Several state or region-specific anti-spitting regulations exist such as in Goa, Mumbai, Delhi, Kerala, Tamil Nadu, Uttarakhand and West Bengal to name a few. Overall, the current law misses out India’s unique use of smokeless tobacco and bans only smoking in public.

**Theme 5.** Health warning on packaging and labelling of tobacco products.

Framework convention treaty	COTPA regulation	Policy gap and qualitative theme
**Article 11.** ‘**….**each unit packet and package of tobacco products and any outside packaging and labelling of such products also carry health warnings describing the harmful effects of tobacco use, and may include other appropriate messages’.	Mandates pictorial and written package warning, in local languages. But unable to regulate tobacco sold through small and domestic trades in rural and semi urban India, in many forms. Most of the package labelling are in English. Warning and information on content of package is misleading due to promotional naming of products attracting different consumer groups.	*‘…majority of the people in this population they don’t know English and the pictorial message is printed in English…. it is either printed in English or Hindi …….so how many people can (understand) when the pictorial message is clubbed with the text message the impact is more so …they can’t read it so sometimes the pictures doesn’t make any sense for the user so those things you know have to be very strongly implemented by the govt. that they have to print the text message in the vernacular language…’ (Professor of Community Oncology, Kerala, India)*

Article 11 of the WHO-FCTC treaty recommends tobacco product packaging guidelines and labelling of packets with information on ill effects as well as contents of the package, to warn consumers [14]. As per COTPA, health warning on tobacco packets, pictorial and written, is supposed to cover 85% of the product package on both sides. In addition, use of information such as ‘light’, ‘ultra-light’, ‘low tar’, etc. is banned as the same is used by tobacco companies as a marketing strategy and misleads the consumers into using tobacco with an assumption that it is less harmful [50]. But due to the availability of unpackaged, loose cigarettes and bidis, made by small manufacturers and also often domestically, many tobacco users do not get to see these package warnings as they buy the cheaper, local variants as well as they buy loose cigarettes. This is more common in rural and semi-urban India where tobacco packets do not bear pictorial warnings. In addition, there are cigarettes available with packets mentioning ‘light’, ‘smooth’, ‘mild’, etc. leading the consumers to believe that they are not being subjected to as much harm [50], and even with pictorial and written warnings, the understanding of these warning images by the illiterate population remains questionable [50]. The Global Adult Tobacco Survey on India indicated that only half of the people (54.7%) were aware of health warnings, and lesser educational attainment was linked to persistent tobacco use as compared to those with higher education attainments. This makes it crucial to re-consider the effectiveness of packaging and labelling of tobacco products on the larger population, who may be uneducated [51]. Another study reported that 25.6% of tribal villagers were aware of pictorial warning but only 21% could interpret the intended meaning [27, 51]. In addition, compliance to WHO-FCTC article 9 describing the need to print tobacco content on packaging is not fully implemented in India due to various indigenous forms of tobacco being produced locally, available domestically through unregulated manufacturing channels [28]. Published data proves that tobacco companies spend a significant proportion of their budget to research on the perception of users on product packaging in order to design new forms of packets to make package labelling ineffective [30].

**Theme 6.** Promotion and advertisement of tobacco products.

Framework convention treaty	COTPA regulation	Policy gap and qualitative theme
**Article 13.** ‘Each Party shall, in accordance with its constitution or constitutional principles, undertake a comprehensive ban of all tobacco advertising, promotion and sponsorship’.	Bans advertisement of tobacco products but cigarette companies continue to use legal loopholes and exercise point of sale advertisement on shops and supermarkets selling tobacco.	*‘…tobacco is injurious to health but you know this hero is smoking (in the movie) so what is the point saying all this because people won’t give that attention, it’s very microscopic also otherwise you have to show in a very big font size but it is so microscopic people won’t give attention…’ (Professor of Community Oncology, Kerala, India)*

Article 13 of the WHO-FCTC treaty recommends restrictions on tobacco advertising and promotion and a comprehensive ban on sponsorships [14]. As per COTPA, advertising through most forms of mass media is banned [16], but it allows point of sale advertisement in shops and does not ban direct and indirect sponsorships by tobacco companies. In collaboration with the Central Board of Film Certification the rules regulate the portrayal of tobacco use in mass media [43, 44]. But the tobacco companies continue to practice surrogate advertisement, brand diversification strategies where they advertise non-tobacco products using the same brand name using loopholes in the law. Tobacco industry still holds a prominent place in the Indian advertising world [35] through these surrogate advertisement and brand diversification strategies. Tobacco companies also conduct targeted promotional campaigns for women, adolescents and youth [27]. A study reported a total sale value for non-tobacco products of a company was INR 67.1 million (US$ 1.45 million) but it spent INR 244.6 million (US$ 5.56 million) in advertising for the same. This proves that ads are made promoting tobacco products the name of non-tobacco products [52]. Overall, inability of the law to regulate these advertisements remains the biggest challenge of current times.

**Theme 7.** Prohibiting production by and sale to minors.

Framework convention treaty	COTPA regulation	Policy gap and qualitative theme
**Article 16.** ‘effective legislative, executive, administrative or other measures at the appropriate government level to prohibit the sales of tobacco products to persons under the age set by domestic law, national law or eighteen’.	Bans selling of tobacco products at certain places and to minors. However, minors continue to be able to access tobacco without any hindrance near schools and colleges. The unregulated part of tobacco industry employs children in huge numbers to manufacture bidis.	*‘bidi rolling industry provides a huge opportunity for subsistence labour and earning so in a way the govt promoted bidi rolling in certain in terms of helping and giving some promotion and that’s why the bidi welfare act also came to give some official support to the bidi labours…’ (Communication Head of an NGO, West Bengal, India)* *‘…..but that factory hardly employs 200 people but they employ 20, 30 thousand 40 thousand people in the backyard and they are invisible and especially when they are children you don’t see them so it’s a huge exploitation thing’ (Communication Head of an NGO, West Bengal, India)* *‘….around 33% of children are girls under 14 who are engaged in bidi labour and basically it starts from around age of 6 below 6 basically nobody rolls bidi…’ (Communication Head of an NGO, West Bengal, India)*

Article 16 of the Framework Convention treaty prohibits sale of tobacco products to and by minors [14]. The Indian law prohibits selling tobacco to minors and bans tobacco sale within hundred yards of educational institutions or through vending machines [16]. It restricts displaying tobacco products at points of sale in the shops and bans minors from handling or selling tobacco products. But the tobacco manufacturing sector, especially the bidi industry, employs children in large numbers. Additionally the sale of loose cigarettes and SLTs attract young users [19, 26, 31]. 625,000 children in India between the age of 10 to 14 use tobacco in some form as per the WHO Tobacco Atlas [10]. Studies have reported that today’s adolescents and youth are able to purchase tobacco products easily within close proximity to their schools and colleges without having to show any proof of age [19, 51]. There are more than 5 million underage users of tobacco in India [31]. Evidence indicates that a younger debut of tobacco leads to a higher likelihood of its sustaining long-term use [31]; the ease with which Indian adolescents are able to access and use tobacco poses a serious threat serious threat to the long-term success of its tobacco control initiatives and regulations.

In addition to this, a major ethical and legal issue imposed by the country’s tobacco industry is employment of children. Out of nearly 5.5 million bidi hand rollers, 85% are women and children [27, 32, 43, 44]. The children work from home without any safety regulation. They are often victims of untreated tobacco exposure related illnesses such as the green tobacco sickness [53]. The current tobacco control law does not specifically address the role of minors working in tobacco industry. It is hard to know the actual numbers of these children due to the informal nature of employment, but estimates range up to one million [54]. Hence, the current Indian law fails to comply with this treaty requirement.

## Discussion

The present paper synthesised qualitative data obtained from experts alongside those obtained from secondary data related to tobacco usage in India. The various themes that emerged from the qualitative data include: ‘Conflicting policies of Government: Promoting tobacco production and at the same time restricting its use’, ‘Failure to shield from influence of tobacco industry’, ‘Demand reduction proposals through taxation and pricing’, ‘Legislation to protect from and reduce the harm of second-hand smoking’, ‘Health warning on packaging and labelling of tobacco products’, ‘Promotion and advertisement of tobacco products’, ‘Prohibiting sale to and by minors’.

The present study had a limitation of being conducted from a single centre, but the authors tried to include views of a of a diverse range of experts from around the country. Due to the sensitive nature of the study question, the study team faced challenges in terms of participant recruitment and applied a purposive sampling approach. It included participants from major metro cities as well as rural India. However, themes generated from the interviews did reach saturation within the limited number of people who were willing to give the interview. The experts were asked about their views and as a result often covered issues in retrospect. The study did not cover emerging issues related to e-cigarettes and did not discuss in detail gaps in tobacco cessation services as well as issues related to Nicotine Replacement Therapy and products. Part of the analysis is based on secondary data, and this has its own limitation being guided by the quality of the primary data that these articles are based on. The figures of the annual turnover of the tobacco industry are based on published consolidated figures of 2010, and more recent update of this could not be found. Strengths of the study include its design adhering to COREC guidelines [24], and the use of robust qualitative methods alongside well accepted frameworks for policy analysis [22] commenting on the tobacco epidemic from a country that has the second highest tobacco consumption globally.

India has played a crucial role in the history of global tobacco trade. The British started growing tobacco in India as a cash crop both for domestic consumption and foreign trade. By 1930, the bidi industry, a smoked form of tobacco, became a significant entity in India and the first cigarette factory, the Indian Tobacco Company was established in 1906 [35]. There were also other chewing forms of tobacco such as ‘gutkha’ which got rapidly commercialised in the past few decades. With the introduction of gutkha and similar other SLT products thereafter, the consumption of chewing tobacco has opened a new battle ground between commercial interests of tobacco players and public health policies in India. Despite legislative measures being in place for decades, due to the uniqueness of its tobacco forms and usage, tobacco control measures have suffered from failure in many aspects due to its inability to address specific issues [25]. Tobacco control in India has come a long way since enactment of the Cigarettes Act in 1975; however, the success of the regulations, supported by several rulings by the Supreme Court against the promotion of tobacco, had suffered due to the lack of implementation and coverage. India continues to reap fiscal benefits from the tobacco trade with an annual turnover value of leading companies exceeding US$ 450 million [50]. In fact, newer varieties of tobacco products have been introduced to the Indian market over the last decade. The Indian tobacco export trend also shows a continuous increase over the last few years [33, 39]. In one hand, increase in taxes has been projected as a measure of public health protection; on the other hand, other governmental departments continued promoting tobacco production by providing subsidies, loans and incentives. Those who use smokeless tobacco in India (163.7 million) are more than double the number of smokers or dual users (42.3 million) and SLTs are the dominant form of tobacco being used in the country [15, 26]. Undermining the importance of the various smokeless forms of tobacco have deterred the success of the overall tobacco control efforts of the country. The smokeless tobacco industry in India is estimated to be worth approximately US$ 1.5 billion and there are around 400 brands of gutkha available in the country [55]. The tobacco industry continues to find legal loopholes to promote smokeless brands as these forms of use are not thoroughly addressed or regulated through COTPA [34]. Several municipal laws enacted in different states attempted to restrict the use of smokeless tobacco, however their success remains a challenge. Internationally, public health advocates have been successful in pressurising tobacco giants to make their tobacco business completely separate [36]. In India, inadequate legislative measures exist to isolate the tobacco brands from the other consumer branding [26]. Many studies have shown that increasing taxes on tobacco are the single most important and cost-effective measure for reduction of demand. In India increasing bidi prices by 10% leads to reduction in rural bidi consumption by 9.2% [13]. There lie tremendous opportunities for the country to increase tobacco taxes for not only cigarettes but for all forms equally as the number of users of smokeless tobacco is much higher in the country. There are significant gaps in addressing early debut of tobacco by the country’s young population.

### Recommended policy reforms

#### Quickly facilitate COTPA Amendment Rules, 2020

The Ministry of Health and Family Welfare, Government of India, published the draft of an Amendment Bill of COTPA and released it in the public domain for obtaining the views of common public. However, the release of this bill is facing resistance from bodies such as All India Farmers Association on account of a drastic loss of employment for the Indian tobacco farmers and a likely increase in illegal tobacco trade. The amended bill proposes to address many of the issues highlighted in this policy analysis such as increasing the minimum age of procuring tobacco from 18 to 21 years of age and stricter implementation of laws against advertising tobacco products. This paper recommends that the following aspects be considered with priority in this amendment bill.

#### 1. Undertake mass level tobacco cessation following the Covid 19 Pandemic

There is an undoubted increased awareness among the common population on harmful effects of tobacco during this COVID-19 pandemic. The first and second wave of the pandemic have experienced imposed restriction on the sale of tobacco products during lockdowns in many states and union territories. Using this changed social viewpoint of tobacco, there is a tremendous opportunity to create mass level awareness on tobacco cessation, stopping of tobacco debut by re-emphasizing the increased risks of morbidity in smokers experiencing Covid 19 infection.

#### 2. Make ‘tobacco-free’ instead of ‘smoke-free’ laws

India is home to the largest number of smokeless tobacco users in the world with 152.4 million SLT users [27]. Bidi and smokeless forms of tobacco manufacturing is the largest tobacco industry in India. Due to its unique burden of smokeless forms of tobacco, the law needs to target controlling different forms of tobacco use in public. Instead of declaring public places smoke free, the law needs to emphasise on achieving tobacco free public places. Alongside cigarettes, banning smokeless tobacco including araca nut and spitting in public places could be looked into by the appropriate authorities. Even the WHO-FCTC acknowledges this gap and makes smokeless tobacco a significant part of their 2025 goals [56]. This paper recommends the government to enact bans on all forms of smokeless tobacco use in public places as well as regulating the supply, procurement and consumption of various other forms of tobacco other than cigarettes.

#### 3. Raise taxes and penalties

In 2014, the Indian government increased the excise duty from 11% to 72%. But the taxation on tobacco products in India is still low and ineffective and the small bidi industries are exempted from paying taxes [57, 58], whereas bidis account for the largest proportion of smoked tobacco in India [27]. It is to be noted that taxes and penalties on tobacco are not adjusted to income growth making tobacco products highly affordable [58]. Hence, one of the recommendations of this paper is to raise taxes for both smoked tobacco and all smokeless forms of tobacco irrespective of origin (handmade or factory made) and stop excise exemption on small scale bidi producers. The revenue from tobacco taxes should be utilised for creating sustainable tobacco control programs.

#### 4. Stop all forms of advertising including point of sale and on packaging

This paper recommends putting a complete ban on all forms of tobacco advertising including on the packets of tobacco products and online advertisements in social media and internet. This includes point of sale advertisements and banning use of the same brand name in any non-tobacco product promotion. Tobacco packets should be designed without any branding and depict the health warnings prominently. This comprehensive ban on advertisement will ensure that tobacco companies have no avenue to reach the consumers with attractive promotions.

#### 5. Ban sale of loose cigarettes and bidis

Unless there is a complete ban on selling loose cigarettes and bidis, the health warnings on tobacco products will not have the intended effect. This paper recommends a total ban on the sale of loose items of smoked tobacco. This will also deter use by the youth and adolescents who may not be able to afford to buy full packets of tobacco.

#### 6. Integrate tobacco control in primary and secondary healthcare and make it mandatory in school education

For an effective and sustainable intervention, tobacco control needs to be integrated in all health and development agendas of the government. This paper recommends integration of tobacco control strategies, especially awareness and cessation support with the primary and secondary level healthcare delivery. Integration of tobacco control with primary healthcare will ensure increased coverage of the rural, uneducated and deprived areas of the country. To achieve this, the National Tobacco Control Program needs to integrate with the primary healthcare delivery in India. Furthermore, mandating tobacco awareness in school curriculums may yield significant impact in terms of prevention of tobacco debut and subsequent use in the young adults. This requires further research.

#### 7. Stop engagement of children in the tobacco trade

Protecting the future generation from the ill effects of tobacco cannot be achieved until there is a strict ban on child labour. This paper recommends revision of the labour law to add strict regulations on children being engaged in any form of tobacco processing either at home or in any industrial setting. Children are the future of the country and providing them with education, a thriving childhood and a safe environment will go a long way to improve the physical and mental health of the country.

## Conclusion

India’s tobacco use behaviour is unique in the world. One of the primary goals of this paper was analysing this uniqueness and understanding the barriers to implementing the policy, instead of just comparing it with global practices. Second, a subject such as tobacco control gives rise to a wide array of political, economic and socio-cultural issue. This report is formulated with a public health lens. It recognises the complex interplay of health issues with its social determinants such as human rights, cultural and economic environment and political will of a country.

In India, 1 million deaths yearly are associated with tobacco use [59]. Tobacco control in India poses its own unique challenges with different forms of use and several barriers in reaching out to the larger population in order to start a cascade of change. The tobacco industry continues to influence all tobacco control efforts. Enormous political will and long-term engagement from a broad range of stakeholders would be required for downsising the tobacco industry. Strict implementation of the law and monitoring the use of tobacco need to go hand in hand. Since a large proportion of cancer in India is tobacco related, we believe that tobacco cessation programmes need to be part of any preventive oncology initiative. Tobacco control needs to be integrated into other government programs from the primary healthcare structure to make it comprehensive and sustainable. This policy analysis highlights the need for revisiting the tobacco control laws in India addressing the existing barriers to have significant impacts on the tobacco picture of this country.

## Funding

All the authors of this paper are in full time employment by Tata Medical Center, Kolkata. There were no external sources of funding for this paper.

## Conflict of interest

The author(s) declare that none of them have any conflict of interest related to this publication.

## Figures and Tables

**Table 1.** Sample characteristics of experts.

Sl. no	Gender	Professional summary	Core area	Research interests
**1**	Female	Public Health Scientist	Prevention of NCDs	Health Promotion
**2**	Female	Founder of a cancer charity working on cancer prevention	Cancer Prevention and Rehabilitation	Quality of Life
**3**	Male	Professor of Community Medicine	Cancer and NCDs	Health Promotion and Community Medicine
**4**	Male	Head and Neck Surgeon	Oral Cancers	Cancer Prevention
**5**	Male	Preventive Oncology Specialist	All Cancers	Cancer Prevention
**6**	Female	Tobacco Cessation Specialist	Head and Neck Cancers	Tobacco Control
**7**	Male	Cancer Surgeon	Head and Neck Cancers	Cancer Treatment
**8**	Male	Leadership role in a charity working with children engaged in Bidi industry	Child Rights Advocacy	Human Rights
**9**	Male	Cancer Prevention NGO and Psychiatrist for a tobacco cessation clinic	Tobacco Cessation	Tobacco cessation

NCDs: non-communicable diseases

**Table 2.** Sources of secondary data included in analysis.

Sl.no	Name of paper	Pub year	Study design	Study population	Paper conclusion	Remarks
**1**	Tobacco Industry Interference Index: Implementation of the World Health Organization’s Framework Convention on Tobacco Control Article 5.3 in India [25]	2020	Desk Review (Review of Literature to assess implementation of article 5.3 of WHO-FCTC in India)	Indian population	Tobacco industry continues to collaborate with government organisations directly or indirectly and continues to influence use of tobacco and public health policies.	Relevant to reemphasize the role of tobacco industry in influencing public health policies and their implementation
**2**	Oral Tobacco and Mortality in India [26]	2016	Narrative Review (Systemic Search of Literature about incidence and prevalence and trend of use of smokeless tobacco (SLT) in Indian men, women, youth, children.)	Indian population	Smokeless tobacco (SLT) use is culturally and historically engrained in Indian population and poses unique challenges in implementing tobacco control measures.	Highlights the uniqueness of India’s tobacco use behavior and the need for customised control strategies
**3**	Assessment of Tobacco Consumption and Control in India [27]	2018	Systematic Review (Review of literature to assess the current tobacco use problem in India)	￼Indian population	Tobacco consumption trend continues to increase in India. It is important to evaluate tobacco consumption patterns to implement suitable solutions of control.	Emphasises on the continued high rates of tobacco use in the country and the need for further analysis of usage patterns to inform policymaking
**4**	Why smokeless tobacco control needs to be strengthened? [28]	2020	Review of Literature (To summarise existing knowledge on use of SLT)	Global population	Population from low socio-economic status is more susceptible to high rates of SLT that is increasing in India. Significant gaps in implementation of law in this regard exist.	India’s SLT use pattern is unique and the same, requiring a different approach of public health legislation, is highlighted in this paper.
**5**	Sociodemographic determinants of tobacco use in India: risks of risk factor—An analysis of Global Adult Tobacco Survey India 2016–2017 [29]	2019	Secondary data analysis of GATS India 2016–2017	Indian Population	Sociodemographic characteristics act as social determinants of tobacco use with education being a strong determinant.	Helps in understanding the tobacco use patterns across a broad sub-section of Indian population.
**6**	Why packaging is commercially vital for tobacco corporations: what British American tobacco companies in Asia tell their shareholders [30]	2017	Analysis of Annual Reports of British American Tobacco (BAT) companies to understand changes in tobacco packaging.	British American Tobacco Companies	Yearly changes in marketing and packaging strategies are done in order to continue tapping consumer markets.	The crucial role of tobacco products packaging and the need to institute stronger policies advocating its restriction are highlighted in this paper.
**7**	Evaluation of a school-based tobacco control intervention in India [10]	2020	Qualitative exploration of perceptions of students, parents, teachers on tobacco use and interventions at school level.	Indian schools	School based interventions have the potential to be affective in changing perceptions of tobacco debut and use. Widespread use of tobacco among students is highlighted.	The need to institute public health policies at school level is reemphasised in this paper.
**8**	In harm’s way: tobacco industry revenues from sales to underage tobacco users in India [31]	2014	Using GATS data to estimate the number of daily underage tobacco users and their annual expenditure on different types of smoked and chewed tobacco products.	Indian Population	Underage daily tobacco users are very high in numbers in the country and their annual expenditure on tobacco products is significant.	Highlights the problem of tobacco use in minors in India and the need for stricter policy reforms to address this issue.
**9**	Health hazards for women in tobacco cultivation in Andhra Pradesh, India [32]	2021	￼Survey and qualitative interviews of women in villages in an Indian state.	Indian village women	Women play a significant role in the cultivation of tobacco but the associated health hazards are ignored.	Focuses on the unregulated segments of tobacco trade and the ethical aspects that need consideration in public health policies.
**10**	A puff of smoke, a hole in the pocket, fissure in the lungs and profit in millions [33]	2019	Case Study on tobacco trade, performance and taxation policies	Indian tobacco companies	Highlights the role large tobacco industries play in terms of their corporate social responsibility and the tradeoff between economic interests and environmental responsibilities.	Highlights the importance of looking at the political-economic interests of the tobacco industry and consider these aspects as key impact generators while devising public health policies.
**11**	Government Interventions on Tobacco Control in India: A Critical Review [34]	2019	Review of effectiveness of anti-tobacco legislations in India based on published and publicly available literature	Indian Population	Implementation of tobacco control law and public health policies need to be more stringent in order to be effective.	Summarises the gaps in regulatory implementation with recommendations well supported by data.
12	WHO Report on Global Tobacco Epidemic [1]	2017	Global report on tobacco control measures, MPOWER strategy, and WHO-FCTC key articles.	Global Population	Data on WHO-FCTC key articles, their importance, strategies to control and prevent use.	Provides a summary of tobacco use and related issues in light of WHO-FCTC.
**13**	The WHO-FCTC: the challenges of implementation [9]	2013	Short communication	Global population	Highlights the challenges faced by the governments of countries in implementing the WHO-FCTC	A comprehensive summary of implementation issues in WHO-FCTC.
14	Perception of tobacco use in young adults in urban India: a qualitative exploration with relevant health policy analysis [19]	2019	Qualitative Study with analysis of health policy	Indian youth	Discusses how the young adults perceive tobacco debut and why they use tobacco, critically analyses what legislative gaps enable the early debut and continued use in youth.	Provides a comprehensive perspective of tobacco behavior and beliefs in youth and highlights the gaps that can be addressed to make regulations effective.
15	Report on Tobacco Control in India (Ministry of Health and Family Welfare Govt. of India website) [35]	2004	Technical Report on Tobacco Use in India	Indian Population	Detailed discussion on tobacco use patterns, practices, health effects, socio economic effects, policies and vision for 2020.	A comprehensive picture of tobacco map of India with recommended future reforms.
16	Conflicts of Interest in tobacco control in India: an exploratory study [36]	2016	￼Exploratory study of documents on tobacco industry available in public domain	Indian tobacco industry	The article found 100 instances of conflicts of interest and then classified these under sub categories; the focus being the government’s conflicting roles and engagements with the tobacco industry.	Very detailed summary of conflicts existing within the government and how the same potentially impact public health policies of tobacco control.
